# Supplementary material for: Population dynamics and seasonal migration patterns of Spodoptera exigua in northern China based on 11 years of monitoring data
Source: PeerJ. 2024 Apr 10;12:e17223. doi: 10.7717/peerj.17223 (PMC11015832; doi:10.7717/peerj.17223)
Supplement: Supplemental Information 1 [file peerj-12-17223-s001.doc]

**Population dynamics and seasonal migration patterns of *Spodoptera exigua* in northern China based on 11 years of monitoring data**

Hao-Tian Ma^1#^, Li-Hong Zhou^2#^, Hao Tan^1^, Xian-Zhi Xiu^1^, Jin-Yang Wang^1^, Xing-Ya Wang^1*^

^1^ College of Plant Protection, Shenyang Agricultural University, Shenyang, Liaoning, 110866, P. R. China

^2^ Institute of Flower, Liaoning Academy of Agricultural Sciences, Shenyang, Liaoning, 110161, P.R. China

^#^ These authors contributed equally to this work

**^*^ Correspondence authors:**

Xing-Ya Wang

120 Dongling Road, Shenhe District, Shenyang, Liaoning, 110866, P.R. China.

Email: [wangxingya20081@syau.edu.cn](mailto:wangxingya20081@syau.edu.cn)

**Supporting Information**


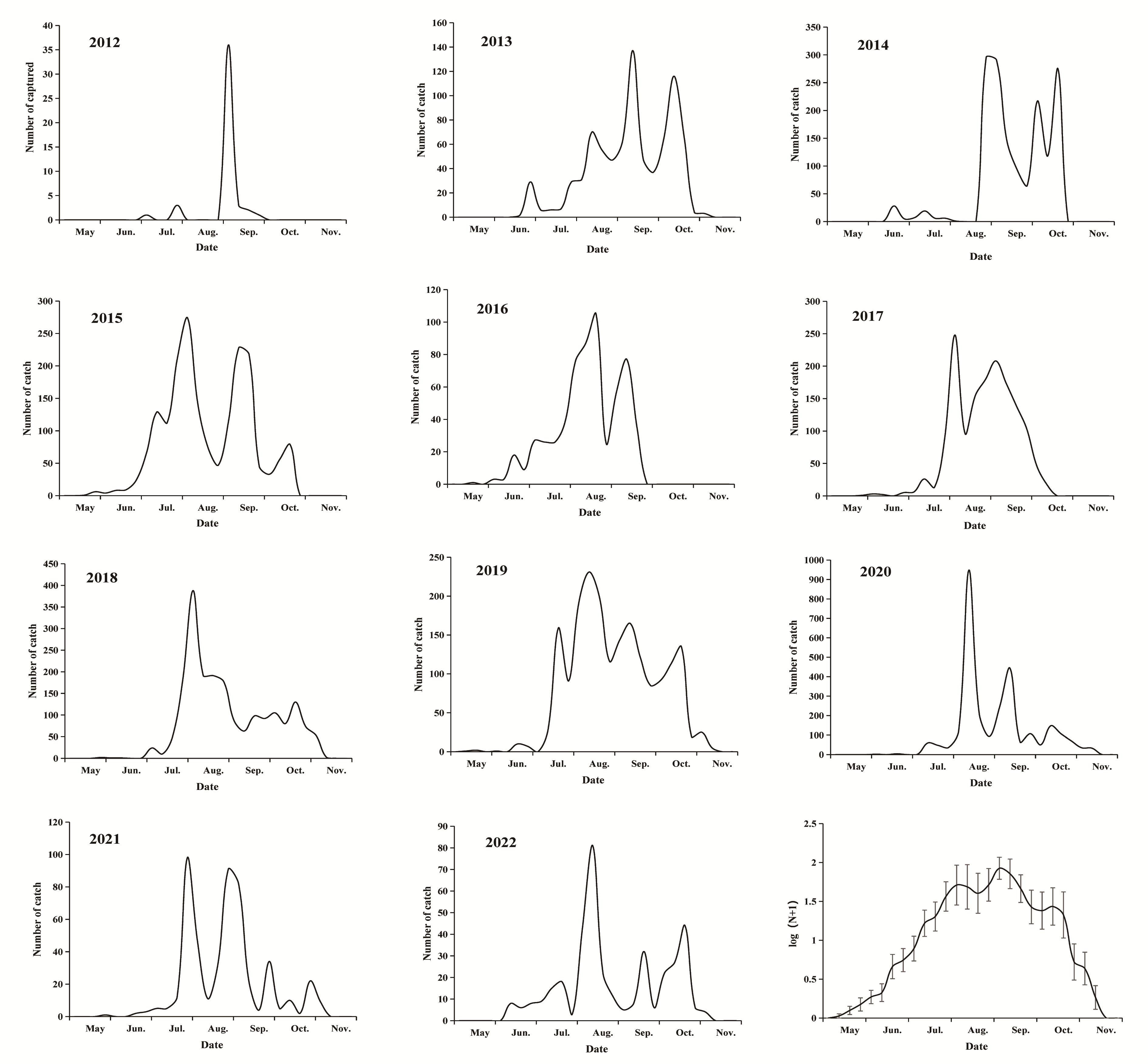


**Figure S1** Population dynamics of captured *Spodoptera exigua* by sex pheromone traps in Shenyang, northern China, during 2012–2022.


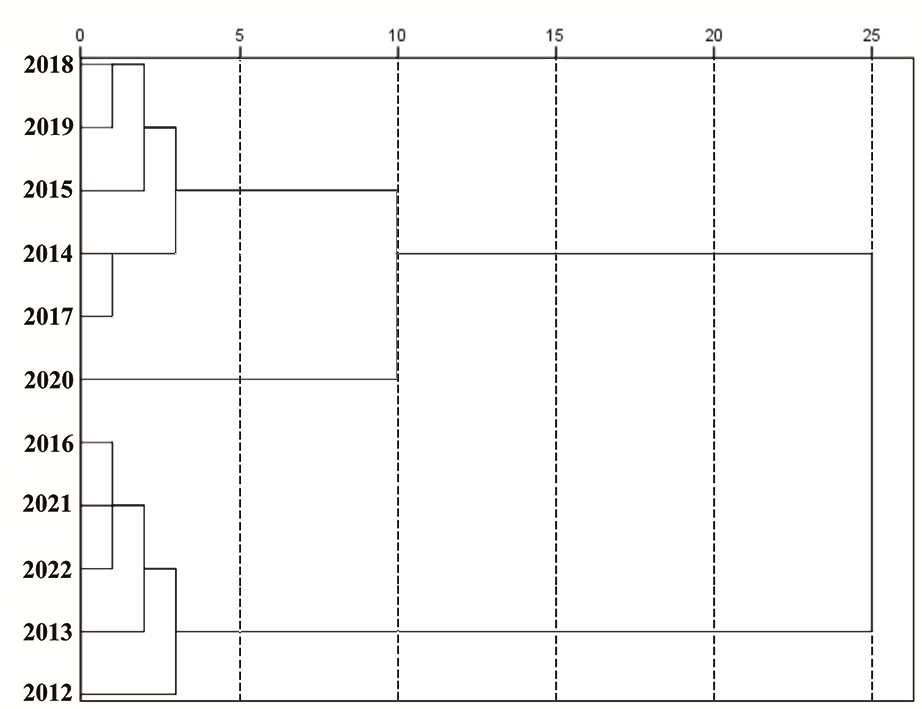


**Figure S2** Clustering dendrogram of *Spodoptera exigua* captured by sex pheromone traps in Shenyang, northern China, during 2012–2022.

**Table S1** Hierarchical clustering analysis of groups on the annual numbers of *Spodoptera exigua* captured by sex pheromone traps in Shenyang, northern China, during 2012–2022.

| **Group** | **Year** | **Observed value**  **(Total captured/year)** | **Distance to center** | **Group mean** | **SD** |
| --- | --- | --- | --- | --- | --- |
|  | 2012 | 46 | 425 | 471.0 | 128.87 |
|  | 2013 | 820 | 349 |
| First group | 2016 | 614 | 143 |
|  | 2021 | 497 | 26 |
|  | 2022 | 378 | 93 |
|  | 2014 | 1576 | 206.4 |
|  | 2015 | 1881 | 98.6 | 1782.4 | 99.91 |
| Second group | 2017 | 1511 | 271.4 |
|  | 2018 | 2002 | 219.6 |
|  | 2019 | 1942 | 159.6 |
| Third group | 2020 | 2828 | 0 |  |  |
| Mean square of cluster | 380646.22 |  |  |  |  |
| Mean square of error | 2423.56 |  |  |  |  |
| *df* | 2, 8 |  |  |  |  |
| *F* | 157.06 |  |  |  |  |
| *P* | 0.000 |  |  |  |  |

**Table S2** The explained variation in meteorological factors and results of significance tests.

| Parameter | Axis 1 | Axis 2 | Axis 3 | Axis 4 |
| --- | --- | --- | --- | --- |
| Explained variation (cumulative) (%) | 47.12 | 54.17 | 58.74 | 59.49 |
| Pseudo-canonical correlation | 0.83 | 0.82 | 0.706 | 0.54 |
| Explained fitted variation (cumulative) (%) | 79.21 | 91.07 | 98.74 | 100.00 |

**Table S3** The explained variation of meteorological factors and signification tests

| Meteorology factors | Explains (%) | Contribution (%) | pseudo-*F* | *P* |
| --- | --- | --- | --- | --- |
| Humidity | 4.80 | 8.10 | 2.40 | 0.096 |
| Wind speed | 43.20 | 72.60 | 18.30 | 0.002 |
| Temperature | 7.20 | 12.10 | 3.30 | 0.020 |
| Rainfall | 4.30 | 7.20 | 2.20 | 0.090 |
